# Risk factors for surgical site infection following hysterectomy: nine-year experience at a large safety-net hospital network in New York City

**DOI:** 10.1017/ash.2025.10153

**Published:** 2025-09-29

**Authors:** Vishal Rana, Tamara Nawar, Temilola-Azeezat Bakare, Jennifer Lee, Olubosede Awoyomi, Mary Fornek, Marie Abdallah, Briana Episcopia, John Quale

**Affiliations:** 1 Department of Infectious Diseases, NYC Health+Hospitals/Kings County, Brooklyn, NY, USA; 2 Department of Infection Prevention, NYC Health+Hospitals/Kings County, Brooklyn, NY, USA; 3 Department of Infection Prevention, NYC Health+Hospitals/Central Office, New York, NY, USA

## Abstract

**Objective:**

To identify risk factors for surgical site infections (SSIs) following abdominal hysterectomy in patients cared for in a large urban public hospital system.

**Design:**

Retrospective case control study.

**Setting:**

Multicenter safety net hospital system.

**Participants all:**

Women undergoing hysterectomy from 2015–2023.

**Methods:**

Propensity score matching, using Centers for Medicare and Medicaid Services (CMS) risk variables, created control groups. Receiver operating characteristics curves were created using current and augmented risk adjustment variables.

**Results:**

There were 6142 hysterectomy surgeries reported during the 9-year time period, with 160 (2.61%) with reportable SSIs. Compared to a matched control group, patients with SSIs were more likely to be of Black race, to have longer duration of surgery, to have open surgery (vs. laparoscopic), and to have received a clindamycin ± gentamicin for surgical prophylaxis. The addition of duration of surgery, endoscopic surgery, and wound class to current CMS risk variables significantly improved the prediction for SSI when all SSIs were included, but did not when patients with superficial SSIs were excluded from analysis.

**Conclusions:**

Predicting SSIs following hysterectomy is complex and current CMS risk assessments are overly simplistic. Until more robust and comprehensive risk assessment criteria are developed, use of SSIs following hysterectomy as a quality measure for reimbursement should be reconsidered.

## Introduction

Over a decade ago, the clinical and financial impact of surgical site infections (SSIs) was recognized.^1^ These infections were associated with increased morbidity and mortality and with increased hospital expenditures ($3.5–$10 billion annually).^1^ For hysterectomies, numerous interventions have been developed, often presented as perioperative surgical bundles, to help reduce the incidence of SSIs. These bundles typically emphasized several features including antiseptic skin prep, appropriate hair removal, and maintenance of normothermia and euglycemia.^2–6^ Many of these features have been included in an updated guidance on SSI prevention.^7^

Since 2015, the Centers for Medicare and Medicaid Services (CMS) has included SSIs following hysterectomies as quality measure that can impact hospital reimbursement. Current CMS risk adjustment variables for abdominal hysterectomy surgery include age, body mass index (BMI), American Society of Anesthesiologists (ASA) score, and the presence/absence of diabetes mellitus. However, several other factors have been found to adversely impact risk of SSIs. Open abdominal hysterectomies carry greater risk then laparoscopic or vaginal surgeries,^8–12^ increased duration of surgery,^8–10^ increased blood loss, contaminated/dirty wound class,^9^ and presence of gynecologic cancer.^9,10^

In this report we examined the rate, and factors associated with, SSIs among patients undergoing abdominal hysterectomies over a 9-year period in a large public healthcare system.

## Methods

The New York City Health and Hospitals System includes 11 public safety net hospitals that serve patients primarily of low socioeconomic status. All are acute care facilities with academic affiliations. Line listings of patients that underwent abdominal hysterectomy surgery from 2015–2023, including those with and without SSIs, were obtained from the National Healthcare Safety Network (NHSN) databank. SSIs were defined according the NHSN criteria.^13^

Propensity score matching was performed to compare surgical risk variables between patients with and without SSIs. The following four CMS risk adjustment variables were included in the propensity score matching: age, body mass index (BMI), diabetes, American Society of Anesthesiology (ASA) score.^14^

Student’s t-test and χ^2^ analysis were used to compare continuous and categorical values, respectively. Propensity score matching was performed using SPSS® to establish a control group of patients without SSIs for the patients with SSIs. To account for the possible impact of technological advancement on SSIs during the nine-year study period, matched cases and controls were obtained on a yearly basis. To identify additional potential risk factors for SSIs, the following information was collected for cases and controls: race/ethnicity, language spoken, NYC borough of residence, presence of uterine cancer, immunosuppressive conditions (HIV, active cancer, immunosuppressive medication), identified alcohol or tobacco use, gravida/para, estimated blood loss, type of abdominal hysterectomy (open, laparoscopic, or robotic), and antibiotic prophylaxis used.

Receiver operating characteristic (ROC) curves, and the corresponding area under the curves (AUCs) for risk adjustment criteria were also determined using SPSS®. AUCs were compared using the methodology described by DeLong et al.^15,16^ For the evaluation of the CMS risk adjustment criteria, all patients with SSIs and patients with deep incisional and organ/space SSIs were included for analysis.

This study was approved by the SUNY Downstate Medical Center Institutional Review Board and the NYC Health and Hospitals Systems to Track and Approve Research program.

## Results

From 2015–2023 there were 6,142 abdominal hysterectomy surgeries reported from the eleven-hospital system. Overall there were 5,982 patients without SSIs and 160 (2.61%) with reportable SSIs following abdominal hysterectomy; the annual rate of infection ranged from 1.45% (in 2016) to 3.84% (in 2022). Of the 160 infections, there were 102 (64%) superficial incisional, 17 (10%) deep incisional, and 41(26%) organ/space infections (20 intra-abdominal, 17 other reproductive tract, and 4 vaginal cuff infections). Twenty-two infections were detected during the same admission, 69 in the outpatient setting, and 69 during a re-admission. Although the 160 patients with SSIs had an average age similar to the 5,982 patients without SSIs (50.4 ± 10.8 vs 49.7 ± 10.5 yr, *P* = NS), the patients with SSIs were more likely to have diabetes (25% vs 15%, *P* = .001) greater BMI (34.4 ± 7.7 vs 30.5 ± 6.5 kg/m^2^, *P* < .001) and ASA score 2.6 ± .6 versus 2.3 ± .6, *P* < .0001). Although the percentage of patients with clean contaminated and contaminated wound classes were similar in each group, there were significantly more dirty wound classes in the patients with SSIs vs patients without SSIs (3 of 160 (1.9%) vs 26 of 5,902 (.4%), *P* = .01). Finally, the duration of surgery was longer in patients with SSIs than those without SSIs (244 ± 100 vs 205 ± 89 mins, *P* < .0001).

Propensity score matching, using the CMS risk adjustment criteria, resulted in 143 matched cases and controls (Table.1). Frequency of alcohol and tobacco use was similar in both groups, as were the percentage of patients with clean contaminated and contaminated wound classes and prior pregnancies. Again, the duration of surgery was significantly longer in the patients with SSIs (Table.1), Significantly more patients with SSIs had open surgical procedures; in contrast, more patients in the control group had laparoscopic surgery. Finally, most patients in both groups received cefazolin±metronidazole; of these patients, 37 of the 99 patients in the control group and 33 of the 85 patients in the SSI received a second intra-operative dose of cefazolin. However, significantly more patients with SSIs received clindamycin ± gentamicin as peri-operative prophylaxis.

**Table 1.** Propensity score matched cases and controls during the nine-year study period

	Controls (n = 143)	SSI Cases (n = 143)	P value
CMS Risk Adjustment Variables			
Age (yrs)	49.6 ± 12.2	50.2 ± 10.9	.65
ASA class (mean±SD)	2.5 ± 0.6	2.5 ± 0.6	.61
BMI kg/m2 (mean±SD)	32.6 ± 6.3	32.7 ± 6.3	.88
DM	31 (22%)	33 (23%)	.78
Other Variables			
Race/Ethnicity (n)			
Asian	17 (12%)	6 (4%)	.02
Black	46 (32%)	65 (45%)	.02
Hispanic	50 (35%)	44 (31%)	.45
White	5 (3%)	7 (5%)	.77
Diagnosis of uterine cancer (n)	35 (24%)	37 (26%)	.79
Immunosuppressive condition or medication (n)	23 (16%)	22 (15%)	.87
Estimated blood loss (ml)	466 ± 672	545 ± 904	.17
Duration of surgery (min)	203 ± 71	257 ± 98	.0002
Surgical technique (n)			
Open	90 (63%)	114 (80%)	.0002
Laparoscopic	34 (24%)	17 (12%)	.009
Robotic	19 (13%)	12 (8%)	.18
Antimcrobial prophylaxis (n)			
Cefazolin±metronidazole	99 (69%)	85 (59%)	.08
Cefoxitin	16 (11%)	14 (10%)	.70
Clindamycin±gentamicin	4 (3%)	16 (11%)	.006
No prophylaxis	13 (9%)	6 (4%)	.10

**Figure 1.**
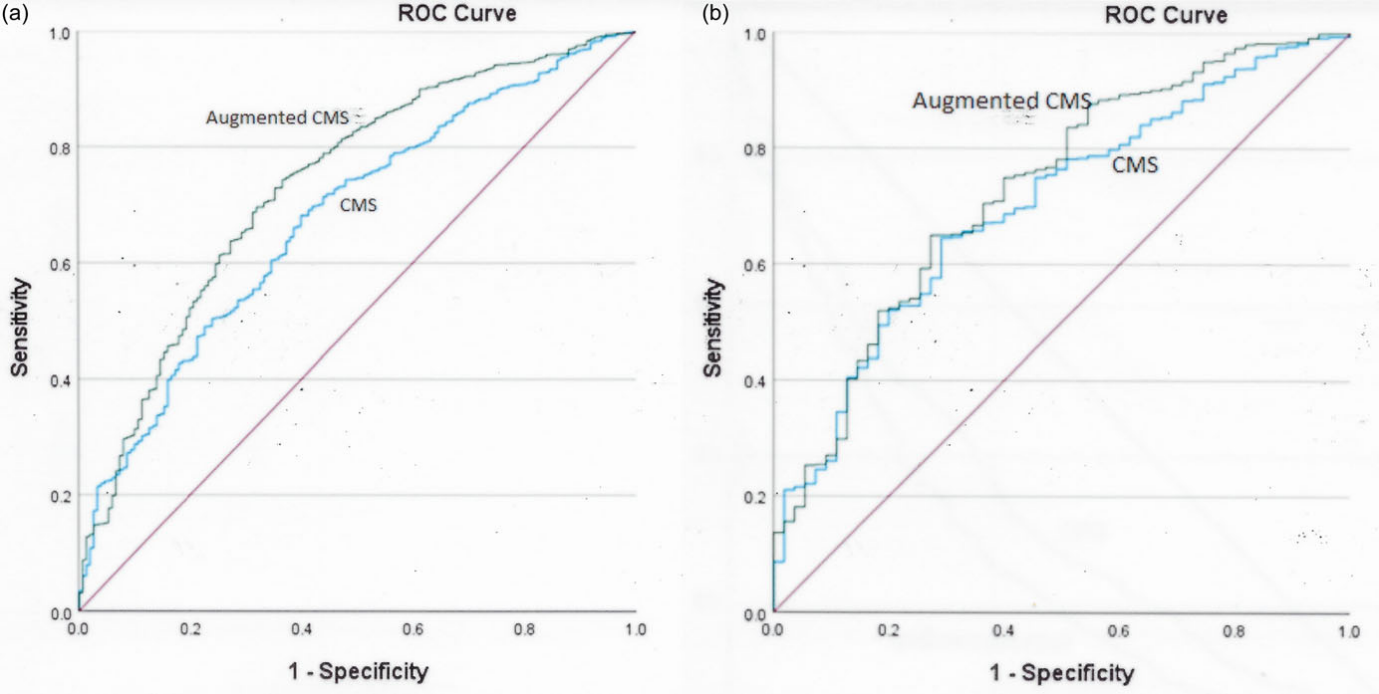
Receiver Operating Characteristic curves for predicting CMS eligible surgical site infections using current CMS risk adjustment criteria (age, BMI, diabetes mellitus, and ASA score) and augmented CMS criteria (addition of duration of surgery, endoscopic surgery, wound class). 1a. All infections included. 1b. Only deep incisional and organ space infections included.

To assess the utility of variables in predicting a SSI, receiver operating curves were developed using the CMS variables (age, BMI, ASA score, and presence of diabetes mellitus) and “augmented CMS” variables (addition of duration of surgery, endoscopic surgery, and wound class). For all SSIs, the augmented CMS performed significantly better than the current CMS variables (area under the curve .735 SE .021 vs .675 SE .021, *P* = .04; Figure 1a). However, when patients with superficial SSIs were excluded from analysis, the area under the curve was only modestly better for the augmented CMS variables compared to the CMS variables (Figure 1b).

## Discussion

Given the clinical and economic consequences, prevention of SSIs following abdominal hysterectomy surgery is being increasingly emphasized in hospitals. An important component of perioperative bundles is the timely administration of appropriate prophylactic antibiotics. Prior studies have prioritized *β*-lactam antibiotics (typically cefazolin) for prophylaxis for abdominal hysterectomy procedures.^17^ Clindamycin with an aminoglycoside is recommended for patients undergoing hysterectomy who have a serious penicillin allergy.^18^ In our report, there were more patients with SSIs who received the recommended alternative prophylactic clindamycin with or without gentamicin compared to matched controls. This finding is likely multifactorial: increasing clindamycin resistance has been reported among staphylococci, streptococci, and anaerobic (eg, Bacteroides spp) bacteria.^19–21^ Also, the average BMI for our patients was >30 kg/m^2^, suggesting suboptimal gentamicin dosing may be a contributing factor. Newer agents, such as tigecycline or eravacycline, should be explored as alternatives for penicillin-allergic individuals. Previous studies have shown that deviations from cefazolin-based prophylaxis may compromise infection prevention efforts,^1,6,7,22^ underscoring the need for robust antimicrobial stewardship programs and accurate allergy documentation.

With the institution of widely accepted bundles, published rates of SSIs following hysterectomy have been varied, ranging from .4%–11%.^4,5,8,12^ Over the final 5 years of our study period, the rate of SSIs was 2.94 ± .61%, and the corresponding standardized infection ratio was 1.260 ±.195 (range 1.035–1.454). We suspect the greater than predicted number of SSIs across our system involves factors beyond the CMS recognized risk factors of age, BMI, ASA score, and diabetes mellitus. Other variables, including some found in our report, that are associated with increased risk of infection include increased duration of surgery,^9,10^ wound class,^9^ and gynecologic cancer.^9,10^ Laparoscopic and vaginal hysterectomies have consistently lower rates of SSIs compared to open abdominal hysterectomies and are the preferred approaches when feasible.^8–12^

Receiver operating curves demonstrated the current CMS risk adjustment variables were suboptimal for predicting SSIs in our population of patients. Adding other risk variables significantly improved the predictive index for all SSIs, but not for the CMS reported deep incisional and organ/space SSIs.

Nearly all the patients treated in the NYC Health + Hospitals system have Medicaid or Medicare as their health insurance, or are uninsured. A recent study documented posthysterectomy infection rates, for both deep incisional/organ space and superficial SSIs, that were nearly double for Medicaid patients when compared to patients with commercial insurance.^23^ In our report, 86% of infections were detected after hospital discharge for the hysterectomy surgery. This is remarkably similar to a recent study by our group, where 90% of infections following cesarean sections in our hospital safety net system were detected following hospital discharge; New York City borough infection rates were directly proportional to poverty level of the borough.^24^ As with cesarean section surgeries, we suspect outpatient wound care following hysterectomy may need to be improved for women of low socioeconomic status.

Several limitations of our study must be acknowledged. First, this was a retrospective analysis relying on surveillance data, which may be subject to underreporting or misclassification. Propensity score matching was used to identify additional factors for infection outside the known CMS risk variables. However, both the infection and control groups were derived from patients of low socioeconomic status attending our safety net hospitals; employing a control group of patients of higher socioeconomic status would likely identify additional factors.

It is apparent that estimating SSIs following hysterectomy is multifactorial and complicated. Current CMS risk assessments for SSIs following hysterectomy have been deemed “inadequate” and do not correlate with other quality summary scores.^25^ The implementation of the Hospital-Acquired Condition Reduction Program and Value-Based Purchasing program, that include hysterectomy SSIs in their measures, have disproportionately penalized safety net hospitals.^26^ The financial consequence of being labeled a “poor performer” is significant, as the CMS penalty of up to 2% of total inpatient charges can be devastating for safety net hospitals. For safety net hospitals, these inaccurate measures of quality may result in augmentation of healthcare disparities.^25^ Until more robust and comprehensive risk assessment criteria are developed, use of SSIs following hysterectomy as a financially-associated quality measure should be reconsidered.
